# “Aggressive” Feeding of Very Preterm Neonates and Body Mass Index at School Age

**DOI:** 10.3390/nu13061901

**Published:** 2021-06-01

**Authors:** Antonios Gounaris, Rozeta Sokou, Martha Theodoraki, Eleni Gounari, Polytimi Panagiotounakou, George Antonogeorgos, Georgios Ioakeimidis, Stavroula Parastatidou, Aikaterini Konstantinidi, Ioanna N. Grivea

**Affiliations:** 1 Neonatal Clinic-NICU, University General Hospital, 41222 Larissa, Greece; ioanna.grivea@gmail.com; 2 Neonatal Clinic-NICU, General Hospital “Agios Panteleimon”, 18454 Piraeus, Greece; sokourozeta@yahoo.gr (R.S.); anastasiosmmr@yahoo.gr (M.T.); ppppolytimi04@gmail.com (P.P.); gantonogeorgos@gmail.com (G.A.); giorgos.ioakeimidis@gmail.com (G.I.); stavroula.parastatidou@gmail.com (S.P.); kmaronia@gmail.com (A.K.); 3 Royal Alexandra Children’s Hospital Brighton, Eastern Road, Brighton BN2 5BE, East Sussex, UK; elenigounari@gmail.com

**Keywords:** very preterm neonates, “aggressive” feeding policies, neonatal period, body mass index, children born preterm

## Abstract

Introduction: The effects of “aggressive” neonatal feeding policies of very preterm neonates (VPN) and the risk of metabolic syndrome later in life remain questionable. We aimed to evaluate the effect of our “aggressive” nutrition policies of VPN during hospitalisation on body mass index (BMI) at ages 2 and 8 years. Materials and Methods: Eighty four VPN, who received “aggressive” nutrition during hospitalisation in an effort to minimise postnatal growth restriction (PGR) (group A), and 62 term neonates, as controls (group B), were enrolled in the study. Group A was further divided in four subgroups depending on the type (A1: fortified expressed breast milk and preterm formula; A2: exclusively preterm formula) and quantity of milk received (A3: maximum feeds 180–210 mL/kg/day; A4: maximum feeds 210 and up to 260 mL/kg/day). BMI was calculated at ages 2 and 8 years and plotted on the centile charts. Results: There was no significant difference in BMI between groups A and B at 2 and 8 years, respectively, in both absolute BMI values and their centile chart distribution. There was no significant difference in BMI at 2 and 8 years either between subgroups A1 and A2 or between subgroups A3 and A4. Conclusions: “Aggressive” and individualised feeding policy for VPN did not affect the BMI and obesity rates at ages of 2 and 8 years in our study population. The type and quantity of milk feeds had no impact on their BMI at school age. Further larger studies are needed to confirm our results.

## 1. Introduction

Since the late 1990s, when intrauterine growth restriction was first linked with metabolic syndrome in adult life [1], there has been increased scrutiny of the feeding policies for very premature neonates (VPN) during their hospitalisation and their effect on postnatal growth.

In the pre-surfactant era, VPN were 3.5 times more likely to develop metabolic syndrome in adult life [2]. A recent meta-analysis, which included preterm neonates from the pre- and post-surfactant era, showed that prematurity is strongly associated with metabolic syndrome in adult life [3].

Metabolic syndrome in adult life is associated with overweight at school age [4]. Determining the body mass index (BMI) at school age can act as a proxy marker for the risk of metabolic syndrome later in life. A recent meta-analysis showed that VPN had significantly increased risk of obesity compared to term controls [5]. To our knowledge, there are limited data regarding the association of VPN BMI at school age with the type and quantity of milk feedings in the neonatal period.

“Aggressive” nutrition, a term introduced by Ziegler in 2002 [6], actually refers to “adequate” nutrition, aiming to reduce postnatal growth retardation (PGR). Such “aggressive” nutrition [6] implemented with variations across different countries and neonatal intensive care units (NICUs), along with the introduction of less invasive respiratory support (early use of nasal continuous positive airway pressure—nCPAP) [7], appears to signify a post-2000 “new era” for neonatology. Nowadays, “aggressive” nutrition focuses on increased protein content, the optimisation of parenteral nutrition, early onset and rapid advancement of enteral feedings, and human milk fortification. Since the introduction of these feeding policies, there was a gradual reduction in the percentage of VPN with PGR from 97% in 1995–1996 [8] to 64.5% in 2000 and 50.3% in 2013 [9]. The question posed by Embleton et al. [10] in 2001—whether PGR was an inevitable consequence of the feeding policies up to that point or there were other possible contributing factors—has only partially been answered, as the percentage of VPN with PGR still remains high.

PGR is associated with short-term morbidity of VPN and adverse late outcomes [11]. In an effort to further reduce PGR, the quality and quantity of milk feedings, the timing of enteral feeding introduction, the rate of feeding advancement, and the optimisation of parenteral nutrition have become a subject of extended discussions among neonatologists, dietitians, and multidisciplinary teams in NICUs over the last few years. There are differing opinions around both the type of milk feeds (expressed breast milk (EBM), use of breast milk fortifier, preterm formula) and the quantity of feeds to achieve optimal growth.

The new feeding policies, in addition to reducing the percentage of neonates with PGR, resulted in better survival rates and improved neurodevelopmental outcomes of VPN in comparison with the pre- and post-surfactant eras of neonatology [12]. Recently, it has been shown that “aggressive” nutrition with different approaches seems to positively affect respiratory outcomes at school age [13,14], but questions remain with regard to its effects on metabolic syndrome later in life.

Furthermore, questions remain regarding the optimal timing for catch up growth of neonates with either intrauterine or extrauterine growth retardation. There is an increased risk of metabolic disturbances for VPN who rapidly increased their weight after 1 year of age [15]. Another study demonstrated that rapid growth (>1 Standard Deviation (SD) for percentile) from 34 weeks to 2 months corrected age correlates with early signs of metabolic syndrome at 6 years of age [16].

In this study, we hypothesised that aggressive feeding, and the subsequent reduction in PGR, may have an impact on BMI at school age, reflecting an effect on obesity risk later in life. The primary aim was to evaluate whether the consistent implementation of our “aggressive” nutrition policy in VPN during hospitalisation has any effect on BMI at ages 2 and 8 years compared to term controls. Additionally, we assessed the effect of the type and maximum amount per kg of milk (EBM or preterm formula) feedings on the BMI of VPN at ages 2 and 8 years.

## 2. Materials and Methods

The initial study population consisted of 84 VPN with birth weight (BW) < 1500 g and gestational age (GA) < 32 weeks admitted to the NICU between 2007 and 2009 who received “aggressive” nutrition according to the unit’s feeding policy and early nCPAP (group A). The control group consisted of 62 term neonates with appropriate weight for gestational age (AGA), hospitalised in our level 1 special care during the study period for minor clinical problems such as feeding difficulties during the first 2 days of life or neonatal jaundice (group B). All neonates with congenital anomalies were excluded from the study.

Study neonates from the two groups were prospectively examined at 8 years of age and had their weight and height recorded and their BMI calculated. Data from the outpatient long-term follow-up files of VPN and personal health records of term neonates were retrospectively collected to calculate BMI at 2 years (referred to as corrected age of 2 years for VPN).

The “aggressive” feeding and respiratory support policies for VPN during their hospitalisation aimed at achieving postnatal growth closely following the Fenton Preterm Growth Chart birth centiles for weight and head circumference (HC), respectively [17]. Our NICU feeding protocol included total parenteral nutrition for all VPN with initial protein content of 1.7–2.5 g/kg/day and final protein content of 3.5–4.5 g/kg/day according to the GA within the first 5 days of life. Fat was added from the second day of life with a final amount of 3 g/kg/day. Milk feeds were introduced from the first or second day of life, mainly maternal milk or, if not available, preterm formula with a calorie content of 81 Kcal/100 mL as trophic feeds for 1–3 days, then increasing the amount with a rate of 24–36 mL/kg/day until achieving full enteral feeding at 200 mL/kg/day. Breast milk fortifier (Nutricia fortifier, protein 1.1 g/100 mL of milk) was added at 100 mL/kg/day of enteral feeds. In case of this being difficult due to clinical disease severity, we still intended to achieve growth as close as possible to birth centiles. Implementation of this policy was guided by weekly evaluation of weight and HC, adjusting the type of milk feedings provided and a gradual increase in the amount of milk feedings up to >210 mL/kg/day in order to achieve growth according to birth centiles.

During hospitalisation, growth was assessed by weekly evaluation of weight gain (g/day) and head circumference (cm/week). Plotting these data on the growth charts on a weekly basis was our guide for the necessary modifications in the feeding schedule of the neonates. This evaluation permitted more accurate monitoring of growth patterns in the chart, which is at present in accordance with recent suggestions by Villar J et al. [18]. VPN continued to receive fortified breast milk or post-discharge preterm formula after their discharge from NICU up until 44 weeks PMA or until HC reached the 25th percentile.

The details about the respiratory support of VPN were recently presented by our research group [14].

Data regarding the type and quantity of milk provided during hospitalisation of VPN were retrospectively collected from hospital files.

Our NICU was at the time the referral regional perinatal centre for neonates born in the West of Attica, Peloponnese and the Aegean islands. Therefore, 65 out of the 84 (77.4%) VPN enrolled in the study were outborn; most of them transported from remote regions. This was the reason for the limited availability of EBM despite our policy supporting breastfeeding.

## 3. Outcome Measures

The BMI of all study neonates at ages 2 and 8 years was expressed as an absolute value and was plotted in the Centers for Disease Control and Prevention (CDC) BMI and BMI Percentile Calculator for Child and Teen [19,20]. We grouped children into categories corresponding to BMI-for-age and sex Percentile Growth Chart: underweight (BMI < 5th centile), normal weight (5th–85th centile), overweight (85th > BMI < 95th centile) and obese (BMI > 95th centile). All VPN were divided into subgroups depending on the type of milk feedings during hospitalisation (subgroup A1 and A2). Study VPN were subsequently divided based on the quantity of milk feedings during hospitalisation (subgroup A3 and A4). BMI was assessed at 2 and 8 years.

Subgroup A1: VPN fed with EBM with fortifier + preterm formula depending on breast milk availability and satisfactory growth. The 16 VPN included in subgroup A1 initially received fortified EBM. In 14 of them, because of poor growth on fortified EBM, we replaced 4–6 out of 12 feeds per day with preterm formula for a period of 10–21 days.

Subgroup A2: VPN fed exclusively with preterm formula.

Subgroup A3: VPN who received 180–210 mL/kg/day of milk feedings.

Subgroup A4: VPN who received >210 and up to 260 mL/kg/day of milk feedings.

In order to investigate how different milk quantities affect growth in VPN, growth rate during hospitalisation was retrospectively calculated using the following formula: body weight at discharge—birth weight/days of hospitalisation.

## 4. Statistical Analysis

Continuous variables are presented as mean (standard deviation) for normally distributed data and median (interquartile range (IQR)) for non-normally distributed data. Categorical variables are presented as absolute and relative values. The assumption of normality was investigated with the implementation of diagnostic plots (Probability–Probability Plots (P–P plots)). T-test was applied in order to examine the association between continuous and categorical variables with two categories, and Pearson’s chi-square test was applied in order to examine the association among categorical variables. All statistical tests are two-sided. The significance level was set at a = 0.05. SPSS statistical software was used for all the study analyses (SPSS Inc. Released 2009. PASW Statistics for Windows, Version 18.0. SPSS Inc.: Chicago, IL, USA). Data analysis was performed from July 2020 to November 2020.

## 5. Results

The study population consisted of 84 VPN with BW < 1500 g and GA < 32 weeks (group A) and 62 AGA term neonates (group B) with BW 3327 ± 405 g (mean ±SD) and GA 39 ± 1.5 weeks (mean ± SD). Demographic and clinical characteristics of the VPN are presented in Table 1.

VPN had milk feedings introduced on day of life 2, (median, interquartile range 2–3), gradually increased to 120 mL/kg/day by day of life 10 ± 4.8 (mean ± SD) and reached full enteral feedings of 150 mL/kg/day by day of life 11 ± 5.2 (mean ± SD). The percentage of VPN < 10th percentile was 7.1% for both BW and HC at birth, while the percentage of VPN < 10th percentile at discharge was 22.6% and 4.8% for body weight and HC, respectively.

VPN did not have significant differences in BMI compared to term controls at 2 and 8 years of age, respectively. In addition, there was no significant difference in the percentage of children classified as underweight, overweight or obese between the two groups (Table 2).

VPN were further subdivided depending on the type and quantity of the milk feedings. In terms of the type of milk feedings, subgroup A1 included 34 VPN and subgroup A2 included 50 VPN.

Regarding the quantity of milk feedings, subgroup A3 consisted of 45 neonates and subgroup A4 consisted of 39 neonates. Comparisons were conducted between subgroups A1 and A2 and subgroups A3 and A4, with no significant difference in BMI at 2 and 8 years of age between the subgroups (Table 3).

The weight gain of VPN during hospitalisation was 20.6 g/day (IQR 17.7–22.93) for subgroup A3 and 21.4 g/day (IQR 19.4–24.97) for subgroup A4 (*p* = 0.34).

## 6. Discussion

There was no significant difference in BMI at 2 and 8 years of age between VPN and term controls in the study population. This applies both to absolute BMI values and their classification based on centile distribution. Neither the type nor the quantity of milk feedings administered to VPN during hospitalisation had a significant effect on BMI at 2 or 8 years.

Children with increased BMI, especially the ones classified as overweight or obese, have increased risk of developing metabolic syndrome later in life [4]. A recent meta-analysis including studies from the post-surfactant era but before the consistent change in feeding policies showed increased risk of obesity for VPN. Furthermore, rapid weight gain up to 2 years of age correlated with increased obesity rates during childhood compared to controls [5]. In our study, the rate of obesity at 8 years of age was 12.7% for the children born VPN compared to 25.9% for those born at term, although this result was not statistically significant. At 2 years of age, 25.7% of children born VPN were underweight, with the rate of obesity remaining extremely low for VPN and terms (1.4% and 2.4%, respectively). Sipola-Leppanen M et al. [21] have previously demonstrated the presence of metabolically active tissues in VPN that were considered protective from the risks of obesity and chronic disease. This can potentially explain the high percentage of VPN classified as underweight at 2 years despite having no significant difference with controls in the studied categories.

In the late 1990s, Lucas et al. showed that neonates who received milk with increased protein content up to 4 weeks after birth had an improved IQ and neurodevelopment at the age of 7.5 years [22]. Recent studies confirmed correlation between PGR and neurodevelopmental and metabolic disturbances in childhood [23,24]. “Aggressive”–“adequate” feeding policies became commonplace after 2000 [6] in an effort to minimise PGR; however, their implementation widely varies. There is ongoing debate with regard to the type and quantity of milk feedings for VPN [25] as well as details including the amount of feedings required to establish full enteral nutrition [25,26]. Despite the variability in its implementation, the central pillars of “aggressive” nutrition—increased protein content, the optimisation of parenteral nutrition, early onset and rapid advancement of enteral feedings, and human milk fortification—have resulted in improved survival and better neurodevelopmental [11] and respiratory outcomes [13,14] for VPN. Further recent studies and a meta-analysis showed that in addition to neurodevelopment, improved nutrition positively affects brain development and growth [27,28,29]. What constitutes optimal feeding for VPN still remains in question, as Menon G et al. noted in their recent article “is preterm nutrition a trade-off between head and heart?” [30].

The optimal type of milk feedings for VPN is still a matter of scientific debate. A systematic review and meta-analysis [31] and a recent study showed that preterm EBM contains 1–1.9 gr/dl of protein during the first month [32]. It is evident that this amount is inadequate for the growth of VPN, especially for the extremely preterm. VPN hospitalised in our NICU received fortified EBM. The number of neonates receiving exclusively EBM was limited in our study due to the significant proportion of neonates transported to our unit from other hospitals. Additionally, the supplementation of EBM with preterm formula was required in a percentage of neonates who failed to achieve “optimal”, as per our assessment, growth on exclusive EBM. Our results showed that the type of milk did not have any effect on BMI at 2 and 8 years of age. This finding could be in accordance with the results of a recently published study by Li et al. [33] comparing VPN receiving EBM feedings, preterm formula and EBM with fortification. This study showed that there was no significant difference in the percentage of adipose tissue at term between the three groups, whereas neonates receiving preterm formula and EBM with fortification had significantly improved weight gain at term. The authors concluded that these results may reflect the lower protein content of EBM.

The maximum quantity of milk feedings is another area of ongoing research and discussion. A recent publication compares amounts of 140–160 mL/kg/day to 180–200 mL/kg/day for premature neonates with BW > 1000 gr to establish the superior one [25]. In the present study, gradually increasing milk feedings of VPN to > 210 and up to 260 mL/kg/day was generally well tolerated without side effects, a result echoed by a similar study where milk feedings were increased up to 300 mL/kg/day in one of the study groups [34]. An increased amount of milk feedings and targeted feeding [25,35,36], as well as not withholding feeds [26], have been shown to decrease PGR to up to <20% of VPN. Recently, Andrews E.T. et al. [36] showed that early postnatal growth failure in preterm infants is not inevitable. In this study, the different amounts of milk feedings in the two subgroups of VPN (180–210 mL/kg/day and >210 mL/kg/day) resulted in similar weight gain during hospitalisation (median 20.6 g/day and 21.4 g/day, respectively), suggesting that similar weight gain can be achieved with different maximum amounts of milk feedings. Furthermore, there was no significant difference in their BMI at 2 and 8 years of age. These results suggest that feeding policies for VPN could be individualised, as each neonate might require different amounts of milk feedings to achieve satisfactory growth. Additionally, in the study population, the targeted and individualised approach on the type and quantity of milk feedings did not adversely affect BMI at school age.

With the implementation of “aggressive” feeding policy during the period 2007–2009 in the current study, the percentage of VPN with weight < 10th centile at discharge was 22.6% compared to a mean of 45.4% for VPN born in 2011/2012 across 19 regions from 11 European countries, ranging up to 60.4% in countries such as Portugal [37]. In addition, in our subjects HC < 10th centile was noted only in 4.8% of the VPN at discharge compared to 7.1% at birth. The International Neonatal Consortium recognised the need to develop new worldwide therapeutic strategies to improve the outcome of preterm neonates [38].

There are limitations to this study. First, it is an observational study with a mainly retrospective design. Providing that the aetiology of obesity is multifactorial, the lack of data such as lifestyle (particularly physical activity) and nutritional habits of enrolled children is another limitation to the study. However, the cultural and socioeconomic homogeneity of our study population might suggest similar behavioural traits. Due to the widespread implementation of “aggressive” nutrition policy in our NICU, there were no less “aggressively” fed neonates for a head-to-head comparison. As data derive from one single NICU, the number of participants is limited, and therefore, results should be interpreted with caution. On the contrary, the consistent implementation of the nutrition policies in a single centre, and the evaluation of the participants’ growth up to 8 years of age, can be considered as the strengths of this study.

## 7. Conclusions

VPN who received “aggressive” nutrition during hospitalisation and up to 40–44 weeks corrected gestation did not show a significant difference in their BMI at school age compared to term controls. Additionally, this feeding policy did not result in a significant increase in the percentage of children classified as overweight or obese. It needs to be further examined whether this result will translate into better metabolic outcomes in the future. In VPN, neither the type nor the quantity of milk feedings significantly affected BMI at school age. These results indicate that preterm feeding can be individualised both in terms of the type and quantity of milk feeds in an effort to optimise growth and minimise PGR. Although the feeding policies implemented in this study were primarily aimed at improving the growth of VPN during hospitalisation, they have not adversely affected BMI at school age. Individualised aggressive/adequate nutrition, based on the neonate’s growth requirements, does not seem to increase the risk of obesity of VPN at school age. Therefore, this feeding policy should be a cornerstone target during the hospitalisation of VPN. Larger prospective studies are needed to confirm these results.

## Figures and Tables

**Table 1:** Demographic and clinical characteristics of study very preterm neonates.

	*n* = 84
Gestational age (weeks.) *	29.1 (2.2)
Birth weight (g) *	1164.5 (299.1)
Days of hospitalisation *	58.3 (29.8)
Respiratory Distress Syndrome (Yes, n (%))	65 (77)
In Vitro Fertilisation (Yes, n (%))	17 (20)
Multiple gestation (Yes, n (%))	37 (44)
Corticosteroids (Yes, n (%))	22 (26)
Intraventricular Haemorrhage grade III–IV (Yes, n (%))	8 (10)
Periventricular leukomalacia (Yes, n (%))	37 (44)
Necrotising enterocolitis (stage IIB–surgical) (Yes, n (%))	1 (1)
Retinopathy of prematurity (Yes, n (%))	29 (35)
Small for gestational age (Yes, n (%))	6 (7)
Sepsis (Yes, n (%))	29 (35)
Bronchopulmonary dysplasia (Yes, n (%))	42 (50)

* mean (standard deviation): Data are presented as mean (standard deviation), or as frequencies (percentages) when appropriate.

**Table 2:** BMI values and children’s classification based on BMI centile distribution at 2 and 8 years.

	2 Years			8 Years		
	VPN	TRM	*p*-Value	VPN	TRM	*p*-Value
BMI (kg/m^2^) (mean (standard deviation))	15.73 (2.06)	15.81 (1.81)	0.833	17.08 (3.51)	18.01 (3.64)	0.119
BMI category	n = 84	n = 62		n = 84	n = 62	
Underweight (%) *	25.7	22.0	0.564	12.0	8.9	0.268
Normal (%) **	61.4	70.7		54.2	46.4	
Overweight (%) ***	11.4	4.9		21.7	19.6	
Obese (%) ****	1.4	2.4		12.7	25.9	
	100	100		100	100	

Abbreviations: BMI, body mass index (calculated as weight in kilograms divided by height in meters squared); VPN, very preterm neonates; TRM, term neonates; * BMI < 5th centile ** BMI 5th−85th centile, *** BMI 85th−95th centile, **** BMI > 95th centile (centiles were recorded using the CDC centile charts for BMI); Data are presented as mean (standard deviation), or as frequencies (percentages) when appropriate.

**Table 3:** BMI distribution of children born VPN according to the type and quantity of milk feeds.

	Study Groups					
	A1 *	A2 **	*p*-Value	A3 ***	A4 ****	*p*-Value
BMI at 2 years (kg/m^2^) (mean (standard deviation))	15.70 (1.71)	15.58 (1.76)	0.775	15.86 (1.82)	15.42 (1.64)	0.294
BMI at 2 years (kg/m^2^) (mean (standard deviation))	17.73 (2.94)	16.67 (3.82)	0.206	16.67 (3.73)	17.42 (3.34)	0.364

* A1: expressed breast milk with fortifier +/− preterm formula ** A2: preterm formula exclusively *** A3: 180–210 mL/kg/day **** A4: 210−260 mL/kg/day; Data are presented as mean (standard deviation).

## Data Availability

The data presented in this study are available on request from the corresponding author.
